# White Matter Microstructural Differences between Hallucinating and Non-Hallucinating Schizophrenia Spectrum Patients

**DOI:** 10.3390/diagnostics11010139

**Published:** 2021-01-19

**Authors:** Justyna Beresniewicz, Alexander R. Craven, Kenneth Hugdahl, Else-Marie Løberg, Rune Andreas Kroken, Erik Johnsen, Renate Grüner

**Affiliations:** 1 Department of Biological and Medical Psychology, University of Bergen, 5009 Bergen, Norway; Alex.Craven@uib.no (A.R.C.); hugdahl@uib.no (K.H.); 2 NORMENT Center of Excellence, Haukeland University Hospital, 5021 Bergen, Norway; else-marie.loberg@helse-bergen.no (E.-M.L.); rune.andreas.kroken@helse-bergen.no (R.A.K.); erik.johnsen@helse-bergen.no (E.J.); 3 Mohn Medical Imaging and Visualization Center, Haukeland University Hospital, 5021 Bergen, Norway; renate.gruner@uib.no; 4 Department of Clinical Engineering, Haukeland University Hospital, 5021 Bergen, Norway; 5 Division of Psychiatry, Haukeland University Hospital, 5021 Bergen, Norway; 6 Department of Radiology, Haukeland University Hospital, 5021 Bergen, Norway; 7 Department of Clinical Medicine, University of Bergen, 5009 Bergen, Norway; 8 Department of Addiction Medicine, Haukeland University Hospital, 5021 Bergen, Norway; 9 Department of Clinical Psychology, University of Bergen, 5009 Bergen, Norway; 10 Department of Physics and Technology, University of Bergen, 5009 Bergen, Norway

**Keywords:** schizophrenia, hallucinations, diffusion tensor imaging, DTI, tract-based spatial statistics, fractional anisotropy, white matter integrity

## Abstract

The relation between auditory verbal hallucinations (AVH) and white matter has been studied, but results are still inconsistent. This inconsistency may be related to having only a single time-point of AVH assessment in many studies, not capturing that AVH severity fluctuates over time. In the current study, AVH fluctuations were captured by utilizing a longitudinal design and using repeated (Positive and Negative Symptoms Scale) PANSS questionnaire interviews over a 12 month period. We used a Magnetic Resonance Diffusion Tensor Imaging (MR DTI) sequence and tract-based spatial statistics (TBSS) to explore white matter differences between two subtypes of schizophrenia patients; 44 hallucinating (AVH+) and 13 non-hallucinating (AVH-), compared to 13 AVH- matched controls and 44 AVH+ matched controls. Additionally, we tested for hemispheric fractional anisotropy (FA) asymmetry between the groups. Significant widespread FA-value reduction was found in the AVH+ group in comparison to the AVH- group. Although not significant, the extracted FA-values for the control group were in between the two patient groups, for all clusters. We also found a significant difference in FA-asymmetry between the AVH+ and AVH- groups in two clusters, with significantly higher leftward asymmetry in the AVH- group. The current findings suggest a possible qualitative difference in white matter integrity between AVH+ and AVH- patients. Strengths and limitations of the study are discussed.

## 1. Introduction

Schizophrenia is a mental disorder that may lead to severe impairments of quality of life and of occupational and social functioning [1,2], and hallucinations, contributing strongly to perceived distress [3,4]. A majority of patients with schizophrenia experience auditory verbal hallucinations (AVH) [5]. The neural underpinnings of the symptoms the patient experiences have been extensively investigated, yet remain unclear. It has been suggested that AVH may involve alterations in structural and functional connectivity of frontal and temporoparietal language-related brain areas [6,7,8] as well as altered internode connectivity within large-scale networks such as the default mode network (DMN) [9,10].

By using diffusion tensor imagining (DTI) to explore microstructural changes in vivo, a substantial number of studies have reported on a correlation between white matter integrity and schizophrenia [11,12,13,14,15,16]. However, the association between AVH, as a key symptom in schizophrenia, and white matter integrity is still not fully understood, and findings are often contradictory. Seok, Park [17] found significant reduction of fractional anisotropy (FA) values when comparing hallucinating (AVH+) and non-hallucinating (AVH-) patients, in the rostral part of the cingulum bundle, the anterior part of the superior longitudinal fasciculus, and the middle cerebellar peduncle, and additionally higher FA-values in the left superior longitudinal fasciculus. In contrast, Ćurčić-Blake, Nanetti [18] found decreased FA-values in the left inferior frontal-occipital fasciculus, uncinate fasciculus, arcuate fasciculus, corpus callosum, cingulate, corticospinal tract, and anterior thalamic radiation in hallucinating patients. Shergill, Kanaan [19], using a voxel-wise approach, showed reduced FA-values in schizophrenia patients vs. healthy controls in several white matter areas, including the right frontal and temporal-parietal portions of the superior longitudinal fasciculus, and the genu of the corpus callosum, as well as in inferior longitudinal fasciculus and tapetum. These authors also found that an increased tendency to hallucinate was related to increased FA-values in the lateral aspects of the superior longitudinal fasciculus and the anterior cingulum.

Several factors could explain the lack of convergence in the current literature. One source of variance is the fluctuating nature of AVH (“state” vs. “trait”) [20,21]. Medication treatment may reduce overt signs of AVH in many patients, such that a “state” assessment could indicate a patient as non-hallucinating, even though he/she has experienced severe and frequent AVHs during earlier periods of the illness, which would be characteristic of a “trait”. Many studies have divided their patients into hallucinating (AVH+) and non-hallucinating (AVH-) sub-groups based on a single assessment (e.g., Seok, Park [17], Psomiades, Fonteneau [22]). As AVHs may have a fluctuating course as a response to treatment [23,24], such approaches may misclassify patients in remission with patients who have never experienced hallucinations. To correctly classify patients as non-hallucinating, multiple assessments over an extended observation-period would therefore be required. In the current study, evaluation of whether a patient would be classified as AVH+ or AVH- was made throughout a 12 month period, using the P3 (third positive item in the scale measuring hallucinatory behavior) item of the Positive and Negative Syndrome Scale (PANSS) [25].

A second issue to be investigated was hemispheric asymmetry and lateralization of white matter tracts. Many studies have shown that schizophrenia patients have altered hemispheric lateralization in comparison to healthy controls. This has been observed in many forms such as handedness [26], grey matter volume [27,28,29], white matter, and functional connectivity [30,31]. Many studies based on DTI have reported reduced left-right asymmetry in schizophrenia patients in white matter tracts related to language processing, e.g., in the arcuate fasciculus [15,32,33], but also bilateral reduction of white matter integrity has been reported [11,18]. However, most of the studies on white matter integrity have not compared values of FA between the left and right hemispheric (e.g., Ćurčić-Blake, Nanetti [18]), have only reported on data from one hemisphere (e.g., Leroux, Delcroix [15], McCarthy-Jones, Oestreich [32]), or limited the analysis to particular fiber tracts (e.g., De Weijer, Mandl [11], Falkenberg, Westerhausen [33]). The current study investigates possible hemispheric asymmetry between groups.

DTI analysis were based on the tract-based spatial statistics (TBSS) approach, which allows for data-driven and bottom-up analysis of white matter structures of the whole brain. The advantage of such an approach is to be able to include the whole brain volume in the analysis, not restricting the analysis to a priori hypothesized structures.

## 2. Materials and Methods

### 2.1. Participants

**Figure 1:**
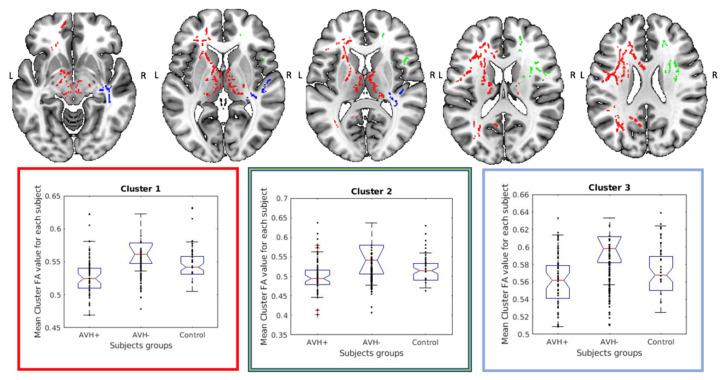
Upper row shows the localization and distribution for the three largest significant clusters, (cluster 1 marked in red, cluster 2 marked in green, and cluster 3 marked in blue) obtained from the comparison between the AVH+ and AVH- groups. The box plot graphs in the lower row shows the corresponding mean clusters Fractional Anisotropy (FA) values for each participant in each group (AVH+, AVH-, Control). The color of the graph frame matches the color of the cluster it belongs to). The central mark indicates the median, and the top and bottom edges of the box represent the 25th and 75th percentiles, respectively. The whiskers extend to the most extreme data points not considered outliers, while the outliers are plotted individually and marked with a plus symbol. Data were merged for the two control groups and presented as a single control group for facilitation of comparison with the patient groups.

**Figure 2:**
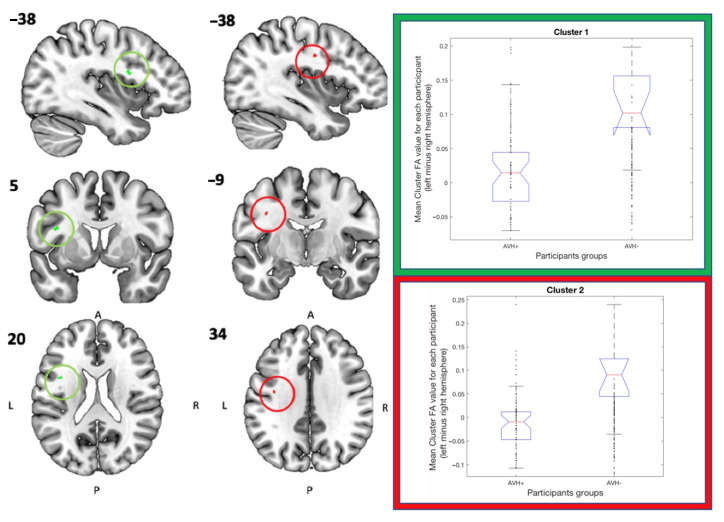
The localization of significant Cluster 1 is marked in green and localization of Cluster 2 is marked in red, see Table 3 for further details regarding localization and size of the two clusters. The box plot graphs represent the corresponding mean asymmetry FA-values (left minus right hemisphere, see *Y*-axis) for each participant in each group. The color of the graph frame matches the colors of the cluster it belongs to. The central mark indicates the median, and the top and bottom edges of the box represents 25th and 75th percentiles, respectively. The whiskers extend to the most extreme data points not considered outliers and the outliers are plotted individually and marked with a plus symbol.

A total of 57 schizophrenia patients were recruited via The Bergen Psychosis Project 2, and compared to 57 healthy controls. The patient and control groups were then subdivided into 44 AVH+ patients and 44 matched healthy controls, and 13 AVH- patients and 13 matched healthy controls, see Table 1 for demographics. Patients were classified as non-hallucinating (AVH-) if they scored lower than “3” on the P3 item on all available assessments for each patient, and patients were classified as hallucinating (AVH+) if they scored “3” or higher at least one out of all available assessments for each patient. For visualization purposes, the two control groups are shown as a single sample of 57 participants in Figure 1 and Figure 2.

The study was approved by the Regional Committee for Medical and Health Research Ethics in Western Norway (REK-VEST, #2010/3387-6). Included patients were 18 years or older with symptoms of psychosis in the schizophrenia spectrum, or with paranoid psychosis according to the International Classification of Diseases ICD-10 diagnostic manual (F20–F29: Schizophrenia, schizotypal, and delusional disorders). All patients were on second-generation antipsychotic medication with average defined daily dose DDD = 0.99 ± 0.58.

Patients underwent psychiatric evaluations at eight time-points during a 12 month period (visit 1—baseline, visit 2—week 1, visit 3—week 3, visit 4—week 6, visit 5—month 3, visit 6—month 6, visit 7—month 9, visit 8—month 12). Due to subject attrition, not all patients were evaluated at all eight time-points, with a median of six assessments. Evaluation over a longer period gave us an overview of the fluctuating character of AVHs for a given patient. The division of the patients into subgroups (AVH+ and AVH-) was based on PANSS P3 scores from all available time-points for each patient. The AVH+ subgroup consisted of 44 hallucinating patients, with a PANSS P3 score of “3” or higher on at least one assessment over the 12 months period. The average PANSS P3 over all visits for the AVH+ group was equal to 3.02 ± 1.66. The AVH- subgroup consisted of 13 non-hallucinating patients (AVH-) with a PANSS P3 score lower than “3” on all available assessments over the 12 months. The average PANSS P3 for AVH- group was equal to 1.13 ± 0.33.

Although the strict selection threshold for AVH- participants resulted in a small sample size, the selection assured that participants were not prone to AVHs, giving us a possibility of comparing hallucinating-prone with non-hallucinating-prone patients. We assumed that patients who experienced AVHs just once and in a mild form, nevertheless had higher chances to have experienced AVHs in the assessment period compared to patients who over the course of 12 months were not experiencing AVHs at all. Here, it is important to mention that although PANSS P3 assesses for hallucinations in a spectrum of modalities (e.g., visual, tactile, olfactory), we used it as a proxy for AVH severity since it has been shown that the auditory modality also dominates in situations with other modalities [34,35,36]. The value of “3” on the PANSS P3 item was chosen as a threshold since, although this indicates “mild” symptom load, such a score is nevertheless indicative of clearly formed hallucinations [25], and is typically used as a cut-off criterion in other studies (e.g., Weber, Johnsen [8]). PANSS ratings were made by certified clinicians/raters, and the number of raters were kept to a minimum, to reduce inter-rater variability. Healthy control participants were recruited from community samples via advertisement in social media and from flyers. The broad announcement was used to obtain a broad representation from the community, i.e., reflecting a randomness that is also believed to be the case for the recruited patients. Among those responding to the advertisements, control participants were continuously blindly selected to best match the recruited patients age, sex, and handedness. The enrollment of control participants was done prior to scanning and was fixed for all the analysis processes and for evaluation of results. No control participants were excluded after scanning. As part of the analysis, the selected control group was further subdivided into two sub-groups: one matched to the AVH+ patients (44 subjects) and one matched to the AVH- patients (13 subjects). Prior to enrollment, the control participants were asked to self-report any history of mental disorder using an in-house questionnaire. A full PANSS evaluation was not performed for these participants as there was no reason to believe that they would provide erroneous answers in the self-reporting on history of mental disorders. Exclusion criterions for the Magnetic Resonance (MR) scanning were history of mental disorders, pregnancy, or standard contraindications to Magnetic Resonance Imaging (MRI), such as any ferromagnetic artefacts in the body. Participants were instructed according to current local hospital practice for all neuroimaging research scanning, i.e., abstain from drinking coffee two hours prior to scanning, to minimize potential confounders. Control participants and patients were included randomly to minimize possible changes in the technical performance of the MR systems. Neither patients nor controls were screened for drugs in urine, although use of medication was registered for both groups.

### 2.2. Image Acquisition

Participants were scanned using a whole-body 3T GE Medical Systems Signa HDx MR scanner at the Department of Radiology, Haukeland University Hospital, Bergen, Norway. The scanner was upgraded to a Discovery MR750 in the middle of the data acquisition period, and a covariate accounting for the scanner version was therefore included in the statistical analysis. As seen in Table 1, there were no significant effects of the scanning upgrade. Imaging data for the matched controls were acquired on the same scanner version as was used for the corresponding patient groups. Head motion was restricted by using foam padding inside the head coil. Diffusion data were acquired with 30 diffusion encoding gradients, b-value 1000 s/mm^2^, and six sets of diffusion unweighted images, phase encoding direction anterior-posterior, FoV 220 mm, TR 14000 ms, TE 84.4 ms, flip angle 90° matrix size 128 × 128 mm, slice thickness 2.4 mm, and total scan time 8.4 min.

### 2.3. Image Processing

The processing pipeline for the DTI-data was performed with FMRIB Software Library (FSL) software package (version 5.0; FMRIB Software Library and the corresponding Diffusion Toolbox). Data were examined visually at all pre-processing steps to identify artifacts as well as to ensure proper data quality. First, a binary brain-mask was created for each subject by using the FSL Brain Extraction Tool (BET) [37]. Second, original data were corrected for head-movements and eddy currents using FSL eddy function with outlier replacement and slice-to-volume outlier correction. We used the eddy function *mporder* to correct for slice-to-volume movements, and the function *repol* to remove any slices judged as outliers and replaced them with predictions made by the Gaussian process. FA-maps were then calculated by using the DTIFit tool, which fits the diffusion tensor model at each voxel. After calculation of the FA-images, voxel-wise statistical analysis of the FA-data was carried out using the TBSS component of FSL [38,39]. This involved alignment of all FA-images onto a common space, using the standard Montreal Neurological Institute (MNI) space atlas, and then subsequently resampling to 1 × 1 × 1 mm^3^ spatial resolution. Thereafter, a mean FA-image was created and further thinned, resulting in a mean FA-skeleton. The skeletonized mean FA-image was thresholded at 0.3 to suppress areas of low mean FA-values, and low and/or high subject-variability. In a next step, the aligned FA-data for all subjects were projected onto the mean FA-skeleton by filling the skeleton with FA-values from the nearest relevant tract center. This resulted in a four-dimensional (4D) image-file which contained the projected, skeletonized FA-data, and which was then entered into statistical analyses. An example of the FA-skeletons is shown in Supplementary Materials Figure S1. For performing hemispheric asymmetry analysis between patients and controls, symmetric mean FA-skeleton images were created. In the analysis, the images were flipped and subtracted from each other to create “left minus right” images using the *tbss sym* function. A detailed description of the *tbss_sym* function is provided in the TBSS manual [40].

### 2.4. Statistical Analysis

In a first statistical analysis, we tested whether voxel-wise FA-values between patient subgroups and their matched control groups were significantly different from each other, with two-sided *t*-tests using the original FA-skeleton. Secondly, significant group-differences in the FA-values between the left- and right hemisphere skeleton were similarly examined, using images from the left-minus-right subtracted symmetrical skeletons. For the first statistical analysis, we performed four group comparisons: (a) comparison between AVH+ and AVH- patients; (b) comparison between AVH+ patients and their matched controls; (c) comparison between AVH- patients and their matched controls, (d) comparison between the two control groups while correcting for sex, age, handedness, and scanner version covariates. The comparison between two control groups was performed to ensure that observed FA-differences between the patient groups were not driven by the differences in sample sizes or influence of covariates of no interest. For the second statistical analysis (FA hemispheric asymmetry analysis), we performed three group-comparisons: (a) comparison between AVH+ and AVH- patients; (b) comparison between AVH+ patients and their matched controls; (c) comparison between AVH- patients and their matched controls, while correcting for sex, age, handedness, and scanner version covariates. Statistical analyses were performed using the permutation interface for general linear model (PALM) [41], which is a tool allowing for inference by using permutation testing. Permutation testing was chosen as it does not impose any assumptions on the distribution of the dependent variables and accounts for possible non-normality of FA-values [42]. All analyses were performed using 10,000 random permutations with threshold-free cluster enhancement (TFCE) [43] with correction of multiple comparisons to a familywise error (FWE) corrected significance level of *p* < 0.05 per analysis. The resulting statistical maps with regions showing significant differences between compared groups and hemispheres were further used for anatomical localization and visualization of the results using the John Hopkins University (JHU) white matter tractography atlas supplied by FSL. A mask of significant regions was created from the images containing p-values, using a cut-off threshold of *p* < 0.05. These clusters served as a mask for the *autoaq* tool supplied by FSL for identification of corresponding anatomical atlas regions. From the significant clusters, the individual FA-values were extracted to illustrate group differences.

## 3. Results

### 3.1. Group Comparison of the Original FA-Skeletons

The comparison of the FA-values between AVH+ patients and their matching healthy controls did not show any significant differences. Similarly, comparison between AVH- patients and their matching healthy controls did not show any significant differences. The additional comparison between two groups of matching controls did not show any significant differences (this was just done to assure that these sub-groups did not differ). However, the comparison between AVH+ and AVH- patients showed widespread and significant reduction in FA-values in the AVH+ subgroup in a number of regions, including bilateral anterior thalamic radiation, bilateral corticospinal tract, bilateral inferior fronto-occipital fasciculus, bilateral superior longitudinal fasciculus, and the temporal part of the right inferior longitudinal fasciculus, forceps minor, and the cingulum bundle. For an overview, see Table 2.

In a next step, we extracted the FA-values from each of the significant clusters found in the AVH+ vs. AVH- comparison to further examine the spatial extent of the findings and perform quantitative comparisons of FA-values, see Figure 1. For all significant clusters, the extracted FA-values were lowest for the AVH+ group and highest for the AVH- group with the controls falling in between. The box plot graphs showing the mean clusters FA-values for each participant in each group (AVH+, AVH-, Control) for all detected clusters is shown in Supplementary Materials Figure S2.

### 3.2. Hemispheric Asymmetry Comparisons

Hemispheric asymmetry analysis was performed using symmetric mean FA-skeleton images, but where the images of the right hemisphere were flipped and subtracted from the corresponding left hemisphere to create “left-minus-right” hemisphere images, using the *tbss sym* function. See Table 3 for co-ordinate localization and size, and the left side of Figure 2 for visualization of the same clusters. The FA asymmetry-analysis showed significantly stronger leftward asymmetry in the AVH- patient subgroup in comparison to the AVH+ patient subgroup in two clusters located in the superior longitudinal fasciculus tract, cfr the bar graphs to the right in Figure 2. As in the preceding analysis, FA-values (here hemispheric differences in FA-values) were lowest for the AVH+ group, highest for the AVH- group, and with the control group non-significantly falling in between.

## 4. Discussion

This study aimed to investigate a possible link between alterations of white matter integrity and auditory verbal hallucinations (AVH). The results showed widespread FA-differences between the AVH+ and AVH- subgroups in several white matter pathways, which have previously been reported to be related to AVH (Bopp, Zöllner [44], Zhang, Gao [45] for examples). In all of the regions with significant findings, we found that patients with AVH had reduced FA-values compared to patients without AVH. In contrast, the FA-values of the healthy control groups were intermediate, although not significantly different from the AVH subgroups. The comparison between two controls groups did not show any significant differences, when controlling for possible confounders. This supports the validity of our findings by confirming that the observed differences for the AVH+ vs. AVH- comparisons were not driven by the difference in sample size or other covariates of no interest between the patient subgroups. Additionally, a significant difference in hemispheric asymmetry was found between the AVH+ and AVH- subgroups in two small clusters in the superior longitudinal fasciculus.

These results partially agree with the findings in the Ćurčić-Blake, Nanetti [18] study, which showed reduced FA-values in some of the major white matter paths, such as: fronto-temporal, cortico-spinal, cingulum, and anterior thalamus tracts and pathways for patients with AVH, see also Oestreich, McCarthy-Jones [16]. On the other hand, a study by Leroux, Delcroix [15] found significantly decreased FA-values for both AVH+ and AVH- subgroups vs. a single healthy control group, but no difference between the AVH+ and AVH- groups. Additionally, they reported FA-reduction in the left arcuate fasciculus when comparing AVH+ subjects to controls, but not when comparing AVH+ vs. AVH-, or AVH- vs. controls. This is in contrast to our findings with regards to the main intra-hemispheric pathways, in particular the inferior fronto-occipital fasciculus. However, both patient selection and data analysis differed between the studies. The studies by Leroux, Delcroix [15], Oestreich, McCarthy-Jones [16], Seok, Park [17], Ćurčić-Blake, Nanetti [18] also found significant differences when comparing AVH+ and AVH- patients to healthy controls, which is in contrast to our study, which showed that the FA-values for the AVH+ group did not differ significantly from the control subjects, while the comparison of AVH+ vs. AVH- groups showed significant differences. The differences between the studies might be that different methods were used to analyze the data and to define the subject groups. Most importantly, neither Oestreich, McCarthy-Jones [16], nor Ćurčić-Blake, Nanetti [18] used healthy control subjects that were matched to their corresponding AVH+ and AVH- patient sub-groups, relying on a single control group. Other differences that could have influenced the results were that the patient sample in our study was characterized by a relatively low mean sample-age, with high variability across age, and illness duration was relatively short.

Aberrations of white matter pathways connecting frontal and temporal brain areas seem to be a frequent finding in DTI-studies of hallucinations in schizophrenia [15,16,18]. Brain structures such as the inferior fronto-occipital fasciculus and the superior longitudinal fasciculus connect frontal and temporal regions, and have essential roles in not only language and semantic speech processing (see, e.g., Duffau, Gatignol [46], Friederici [47], Glasser and Rilling [48], Rilling, Glasser [49], but also in attention, working memory, and emotions [50,51]. Hugdahl, Løberg [7] hypothesizes that the dynamic interplay between top-down (information processing) and bottom-up (stimulus processing) might be unbalanced in AVHs. Moreover, he suggested that intact and connected prefrontal and temporal areas might be the reason why healthy voice-hearers can resist being overwhelmed by their “voices” in comparison to schizophrenia patients suffering from AVH. Our finding of decreased FA-values in fronto-temporal and fronto-occipital pathways support the model proposed by Hugdahl, Løberg [7] since the aberrations of white matter pathways connecting frontal and temporal lobes might influence information flow from frontal cortex, which in turn might result in failure to inhibit bottom-up temporal lobe hyper-activity. We also observed abnormalities in the anterior thalamic radiation and in the cingulum bundle. Both of these pathways have been shown to play a role in emotion processing [52,53,54,55], a modality which is central in AVHs since AVHs typically are negatively charged. One could hypothesize that since we did not find significant differences for PANSS total negative scores between the patient groups, abnormality of these white matter tracts might also influence the emotional content of AVH.

In a study by Miyata, Sasamoto [56], schizophrenia patients exhibited overall reduced asymmetry compared to healthy controls. Following this, the current results with significantly reduced leftward FA asymmetry in the AVH+ group compared to the AVH- group, could mean that AVHs add to the asymmetry reduction seen in schizophrenia in general. Other studies have shown leftward asymmetry of the planum temporale and auditory cortex at the back of the superior temporal gyrus in healthy populations, which has been linked to left hemispheric functional specialization for language [57,58]. Furthermore, leftward asymmetry of the anterior cingulum and the arcuate fasciculus, the corticospinal tract and the posterior limb of the internal capsule has been reported in studies of white matter hemispheric asymmetries in healthy individuals [59]. Therefore, it may be suggested that reduced leftward asymmetry in our AVH+ patents for major white matter pathways connecting frontal and temporal brain areas may be related to dysfunction of language areas in AVH. It has been previously shown that parts of the frontal superior longitudinal fasciculus, which contain the arcuate fasciculus, also connect to the frontotemporal language association cortex [60]. Several studies suggest that the left arcuate fasciculus, which is the critical pathway of the language network, show reduced FA-values in AVH+ patients [6,17,61,62]. However, none of the previous studies have demonstrated a direct link between hemispheric differences for FA-values, and for difference in asymmetry between AVH+ and AVH- patients, indicating a structural correlate to known behavioral and functional differences between AVH+ and AVH- patients (cf. Falkenberg, Westerhausen [33], Hugdahl, Løberg [63]).

Some limitations of the present study deserve discussion. Firstly, patients underwent treatment with antipsychotic medication throughout the whole duration of the study, which is always a source of confounding. However, we did not see differences between the AVH+ and AVH- groups in DDD values, which would indicate that medication differences may not have been a major source of confounding of the results. A second limitation relates to the use of DTI as a general measure of white matter integrity and functionality. It is important to remember that although FA-values carries information about white matter-state at the cellular level, this information is averaged over large voxel volumes since the MRI resolution is much larger (mm) than the scale of the cells that are probed by diffusing water (μm) size [64]. The presence of, for example, multiple fiber populations with different fiber orientations may also contribute to the average value of the signal. Therefore, while observing decreased FA-values in specific brain regions, it cannot be assumed that this exclusively results from abnormalities at the cellular level. It could as well be due to the reorganization of the fibers on a macroscopic scale. It can also be the combination of changes in tissue myelination, fiber organization, as well as the number of axons [65]. Therefore, the presented results should be interpreted with caution. It should be kept in mind that FA is a general measure of white matter integrity and observed changes can be of the multifactorial origin (e.g., cell death, edema, gliosis, inflammation, change in myelination, increase in connectivity of crossing fibers, increase in extracellular or intracellular water, etc.) [64]. It should also be mentioned that parts of the patient data included in the current study were used in the [33] study as well as [66]. However, the composition of the samples in the studies was different, as well as the hypotheses, methods, and analysis technology used.

## 5. Conclusions

In conclusion, the most consistent finding was the alteration of hemispheric language pathways in AVH+ patients, which has also been reported in previous studies, although the nature and direction of this relation have often differed [6,15,16,19]. Interestingly, we also found a significant difference between AVH+ vs. AVH- patients, but not for the comparison with the healthy controls. This could be an indication that non-hallucinating schizophrenia patients form a distinct subgroup from the schizophrenia spectrum. This in turn, could mean that differences between AVH+ and AVH- patients should be studied more thoroughly in the future since it might have implications for the choice of treatment options for patients with severe and persistent hallucinations. The present study provides evidence for the importance of translational studies, bridging the gap between neurobiology and psychiatry, especially for major mental illnesses [67].

## Figures and Tables

**Table 1:** Demographic and clinical information for the three groups (hallucinating patients (AVH+), non-hallucinating patients (AVH-), and healthy controls, (Cntrl)).

	AVH+	AVH-	Cntrl	*p* (Value)
Sample size (female/male)	44 (12/32)	13 (2/11)	57 (15/42)	0.72 Fisher’s Exact test
Age (mean ± SD) ^a^	30.1 ± 11.4	29.5 ± 9.3	30.7 ± 9.9	0.92 ANOVA
Handedness (A/L/R) ^b^	2/4/38	1/1/11	0/6/51	0.35 Fisher’s Exact test
Scanning after/before the upgrade	21/23	5/8	25/32	0.86 Fisher’s Exact test
Duration of illness	3.6 ± 4.7	2.2 ± 4.4	-	0.40 Welch Two Sample *t*-test
Defined Daily Dose (DDD)	1.02 ± 0.63	0.91 ± 0.41	-	0.51 Welch Two Sample *t*-test
Smoking/non-smoking	28/16	10/3	-	0.51 Fisher’s Exact test
Baseline PANSS ^c^ positive	3.0 ± 0.7	2.6 ± 0.7	-	0.04 Welch Two Sample *t*-test
Baseline PANSS negative	2.4 ± 0.8	2.0 ± 0.7	-	0.99 Welch Two Sample *t*-test
Baseline PANSS total	2.7± 0.6	2.3 ± 0.6	-	0.03 Welch Two Sample *t*-test

^a^ SD—Standard deviation, ^b^ A—Ambidextrous, L—Left handed, R—Right handed, ^c^ PANSS—Positive and Negative Syndrome Scale.

**Table 2:** Localization and size of significantly different clusters between AVH+ and AVH- participants. The numbers after each tract label indicates the percentage probability of the cluster to belong to the given (John Hopkins University) JHU white matter tractography atlas label.

Number of Voxels	X (mm) ^a^	Y (mm)	Z (mm)	Major Tracts Included in a Cluster ^b^
13231	−36	−1	28	Anterior thalamic radiation L ^c^: 5.9 Anterior thalamic radiation R: 2.7 Corticospinal tract L: 1.8 Corticospinal tract R: 1.0 Forceps minor: 2.0 Inferior fronto-occipital fasciculus L: 1.4 Superior longitudinal fasciculus L: 2.2
1752	32	7	28	Anterior thalamic radiation R: 1.2 Forceps minor: 3.8 Superior longitudinal fasciculus R: 6.4 Superior longitudinal fasciculus (temporal part) R: 2.4
948	45	−22	5	Inferior fronto-occipital fasciculus R: 14.4 Inferior longitudinal fasciculus R: 9.8
530	34	−59	29	Superior longitudinal fasciculus R: 7.5 Superior longitudinal fasciculus (temporal part) R: 1.3
295	17	−52	25	Cingulum (cingulate gyrus) R: 1.3
275	12	4	−7	Anterior thalamic radiation R: 14.8
89	49	−4	−12	Inferior longitudinal fasciculus R: 6.8
53	31	−32	37	Superior longitudinal fasciculus R: 16.3
46	24	−54	24	Inferior fronto-occipital fasciculus R: 5.0 Inferior longitudinal fasciculus R: 1.7 Superior longitudinal fasciculus R: 1.3
38	34	−11	−5	Inferior fronto-occipital fasciculus R:3 9.4 Inferior longitudinal fasciculus R: 1.2
30	36	−4	−5	Inferior fronto-occipital fasciculus R: 8.7 Superior longitudinal fasciculus R: 3.8 Superior longitudinal fasciculus (temporal part) R: 2.3
25	23	−50	32	Anterior thalamic radiation R: 1.9 Inferior fronto-occipital fasciculus R: 2.9 Superior longitudinal fasciculus R: 1.6
15	19	−47	35	Cingulum (cingulate gyrus) R: 4.2

^a^ MNI—Montreal Neurological Institute; ^b^ values reflect the percentage probability of the cluster to belong to the given atlas label; ^c^ L—abbreviation for the left hemisphere, R—abbreviation for the right hemisphere.

**Table 3:** Montreal Neurological Institute (MNI) space co-ordinates, number of voxels, and major white matter tracts included in the two significant clusters, labeled Cluster 1 and Cluster 2, respectively. The numbers after each tract label indicates the percentage probability of the cluster to belong to the given JHU white matter tractography atlas label.

Cluster Index	Number of Voxels	X ^a^ (mm)	Y (mm)	Z (mm)	Major Tracts Included in a Cluster ^b^
1	22	−38	5	20	Superior longitudinal fasciculus: 13.2 Superior longitudinal fasciculus (temporal part): 6.9 (marked in green on Figure 2)
2	20	−38	−9	34	Superior longitudinal fasciculus: 15.4 Superior longitudinal fasciculus (temporal part): 7.0 (marked in red on Figure 2)

^a^ MNI—Montreal Neurological Institute; ^b^ values reflect the percentage probability of the cluster to belong to the given atlas label.

## Data Availability

The datasets analyzed during the current study are not publicly available. According to Norwegian law, data sharing requires approvals from the Regional Committees for Medical and Health Research Ethics, and from the Data Protection Officer at Haukeland University Hospital, on the basis of specific research proposals.

## Supplementary Materials

The following are available online at https://www.mdpi.com/2075-4418/11/1/139/s1, Figure S1: mean FA-skeleton masks derived for three comparisons, Figure S2 The box plot graphs showing the mean clusters FA-values for each participant in each group (AVH+, AVH-, Control).

## Abbreviations

4D	Four Dimensional
ANOVA	Analysis of Variance
AVH	Auditory Verbal Hallucinations
BET	Brain Extraction Tool
DDD	Defined Daily Dose
DMN	Default Mode Network
DTI	Diffusion Tensor Imaging
ERC	European Research Council
FA	Fractional Anisotropy
FMRIB	Functional Magnetic Resonance Imaging of the Brain
FSL	FMRIB Software Library
FWE	Familywise Error
GE	General Electrics
HD	High-definition
ICD	International Classification of Diseases
P3	Positive three
PANSS	Positive and Negative Syndrome Scale
SD	Standard Deviation
TBSS	Tract Based Special Statistics
TE	Echo Time
TFCE	Threshold Free Cluster Enhancement
TR	Repetition Time
