# Supplementary material for: White Matter Microstructural Differences between Hallucinating and Non-Hallucinating Schizophrenia Spectrum Patients
Source: Diagnostics (Basel). 2021 Jan 19;11(1):139. doi: 10.3390/diagnostics11010139 (PMC7832406; doi:10.3390/diagnostics11010139)
Supplement: Supplementary file 1 [file diagnostics-11-00139-s001.pdf]

Supplementary materials

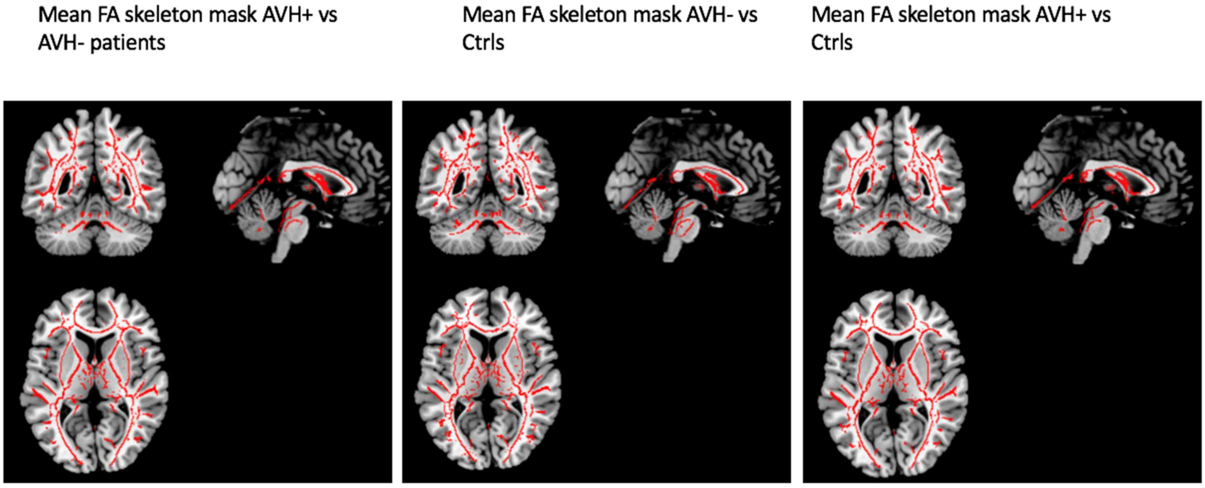

(a) (b) (c)

Figure S1. Mean FA skeleton masks derived for three comparisons: a) hallucinating schizophrenia patients (AVH+) vs. non-hallucinating patients (AVH-); b) non-hallucinating schizophrenia patients vs. matched heathy controls; c) hallucinating schizophrenia patients vs. matched healthy controls.

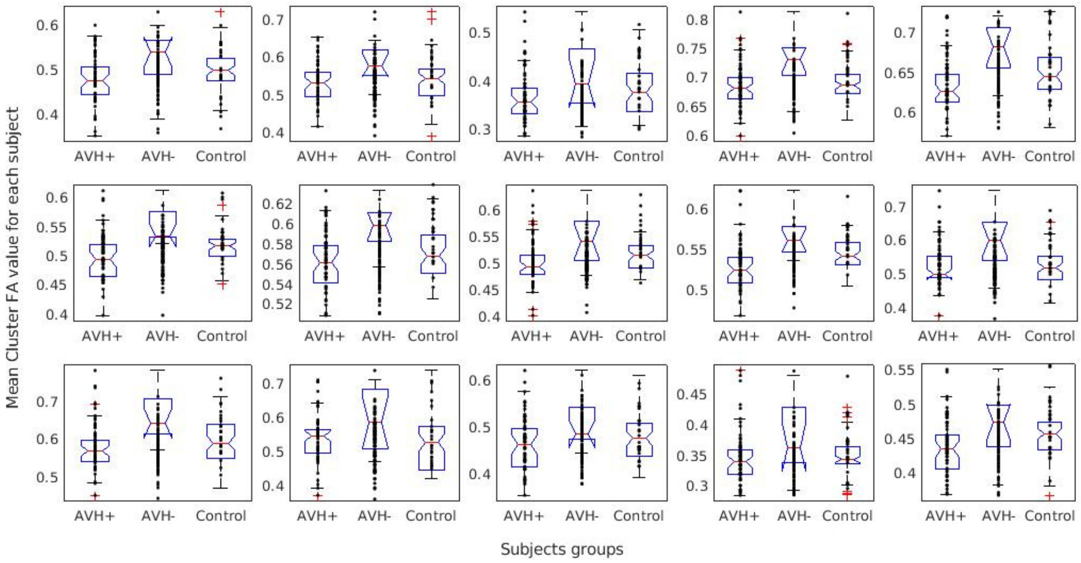

Figure S2. The box plot graphs showing the mean clusters FA values for each participant in each group (AVH+, AVH-, Control). The central mark indicates the median, and the top and bottom edges of the box represent the 25th and 75th percentiles, respectively. The whiskers extend to the most extreme data points not considered outliers, while the outliers are plotted individually and marked with a plus symbol. NB. Data were merged for the two control groups and presented as a single control group for facilitation of comparison with the patient groups.
